# Draft Genome Sequence of *Xylella fastidiosa* subsp. *multiplex* Strain Griffin-1 from *Quercus rubra* in Georgia

**DOI:** 10.1128/genomeA.00756-13

**Published:** 2013-10-10

**Authors:** Jianchi Chen, Hong Huang, Chung-Jan Chang, Drake C. Stenger

**Affiliations:** USDA Agricultural Research Service San Joaquín Valley Agricultural Sciences Center, Parlier, California, USAa; School of Information, University of South Florida, Tampa, Florida, USAb; Department of Plant Pathology, University of Georgia, Griffin, Georgia, USAc

## Abstract

The draft genome sequence of *Xylella fastidiosa* subsp. *multiplex* strain Griffin-1, isolated from a red oak tree (*Quercus rubra*) in Georgia, is reported here. The bacterium has a genome size of 2,387,314 bp, with a G+C content of 51.7%. The Griffin-1 strain genome contains 2,903 predicted open reading frames and 50 RNA genes.

## GENOME ANNOUNCEMENT

*Xylella fastidiosa* is a Gram-negative, xylem-limited plant-pathogenic bacterium (1) causing many economically important diseases, including oak leaf scorch disease in the eastern United States (2–4). Due to nutritional fastidiousness, characterization of *X. fastidiosa* has been difficult, and many biological issues related to *X. fastidiosa* strains remain to be investigated. Both complete genome (5–8) and whole-genome shotgun (9, 10) sequences of *X. fastidiosa* are currently available. Three subspecies of *X. fastidiosa* (subspecies *fastidiosa*, subspecies *multiplex*, and subspecies *pauca*) have been proposed (11). The taxonomic statuses of *X. fastidiosa* strains from oak trees have not been evaluated. An early study based on a random amplified polymorphic DNA analysis showed that *X. fastidiosa* strains from turkey oak (*Quercus laevis*) in Florida and red oak (*Quercus rubra*) in Georgia were highly similar (12).

In the summer of 2006, a strain of *X. fastidiosa* was isolated from a symptomatic red oak tree (*Q. rubra*) in Griffin, Georgia (33°15′38.07″N, 84°16′48.69″W), and it has been maintained in our laboratory in California. This bacterial strain was triple cloned and designated Griffin-1. To obtain genomic DNA, Griffin-1 was cultured in periwinkle wilt (PW) broth (13) at 28°C for 10 days. Bacterial cells were collected by centrifugation; the total genomic DNA was extracted by a standard procedure (14). Genome sequencing was carried out on a 454 GS-FLX system using Titanium chemistry (Roche) (15). Paired-end reads were assembled with the Newbler software (Roche Diagnostics). The Griffin-1 genome consists of 2,387,314 bp (~30× coverage, G+C content of 51.7%) assembled into 84 contigs ranging from 523 bp to 142,581 bp. Annotation was performed by the RAST server (http://rast.nmpdr.org/) (16), which utilizes the GeneMark, Glimmer, and tRNAscan-SE databases. The Griffin-1 genome was predicted to have a total of 2,903 open reading frames (ORFs) and 50 RNA genes.

Using BLAST analyses (17), the sequences of *ssr* (16S rRNA) and four housekeeping genes, *gyrB* (DNA gyrase subunit B), *dnaK* (chaperone protein), *rpoD* (RNA polymerase sigma factor), and *tonB* (outer membrane receptor), were selected and compared to the corresponding gene sequences of *X. fastidiosa* subsp. *multiplex* strain M12 (5), *X. fastidiosa* subsp. *fastidiosa* strains M23, GB514, and Temecula (5, 6, 8), and *X. fastidiosa* subsp. *pauca* strain 9a5c (14). For all five loci, *X. fastidiosa* subsp. *multiplex* Griffin-1 is 100% identical to strain M12, indicating that the oak strain is a member of *X. fastidiosa* subsp. *multiplex.*

### Nucleotide sequence accession numbers.

This whole-genome shotgun project has been deposited at DDBJ/EMBL/GenBank under the accession no. AVGA00000000. The version described in this paper is version AVGA01000000.
